# Supplementary material for: Deletion of EP4 in S100a4-lineage cells reduces scar tissue formation during early but not later stages of tendon healing
Source: Sci Rep. 2017 Aug 17;7:8658. doi: 10.1038/s41598-017-09407-7 (PMC5561254; doi:10.1038/s41598-017-09407-7)
Supplement: Supplementary file 1 — Supplemental Figures [file 41598_2017_9407_MOESM1_ESM.pdf]

Deletion of *EP4* in *S100a4*-lineage cells reduces scar tissue formation during early but not later stages of tendon healing

Jessica E. Ackerman[1], Katherine T. Best[1], Regis J. O'Keefe[2], Alayna E. Loiselle[1,*]

[1]Center for Musculoskeletal Research, University of Rochester, Rochester, New York, United States of America
[2]Department of Orthopedic Surgery, Washington University School of Medicine, St. Louis, Missouri, United States of America

*Corresponding Author
Email: Alayna_Loiselle@urmc.rochester.edu

Supplemental Materials

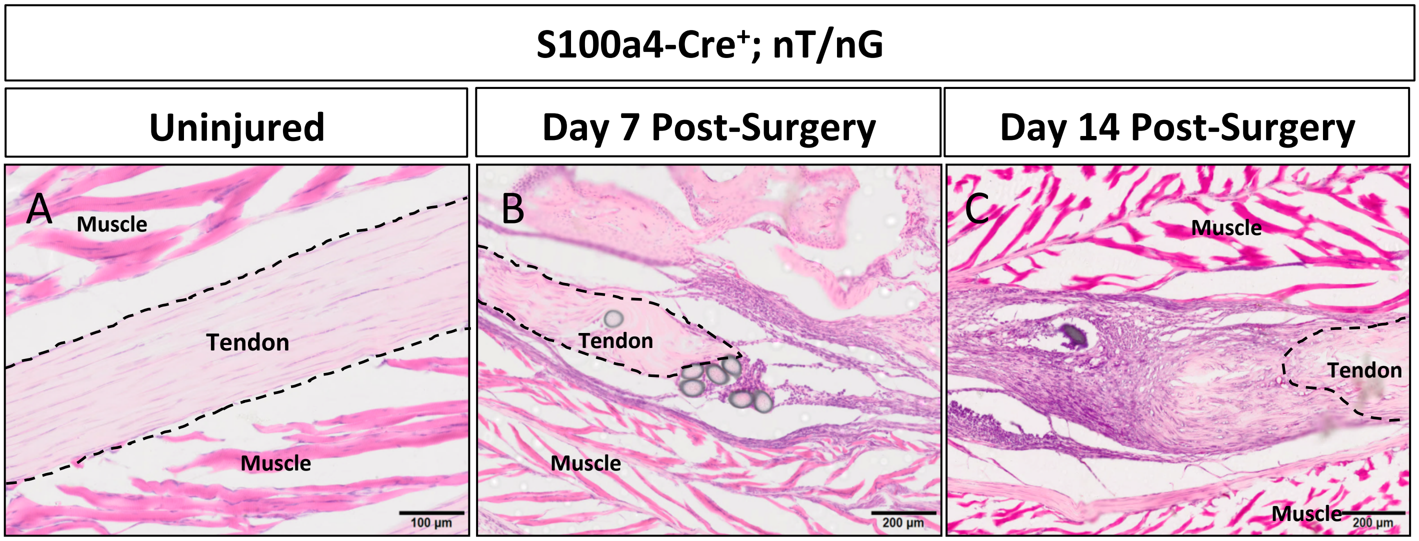

Supplemental Figure 1. Hematoxylin & Eosin (H & E) staining of (A) un-injured, and repaired tendons at (B) 7, and (C) 14 days post-surgery from *S100a4*-Cre^{+}; nT/nG mice. Following fluorescent imaging (Figure 1), coverslips were gently removed and sections were stained with H&E to assess tissue and cell morphology. Tendon is outlined in black. Scale bars represent (A) 100 microns, (B&C) 200 microns.

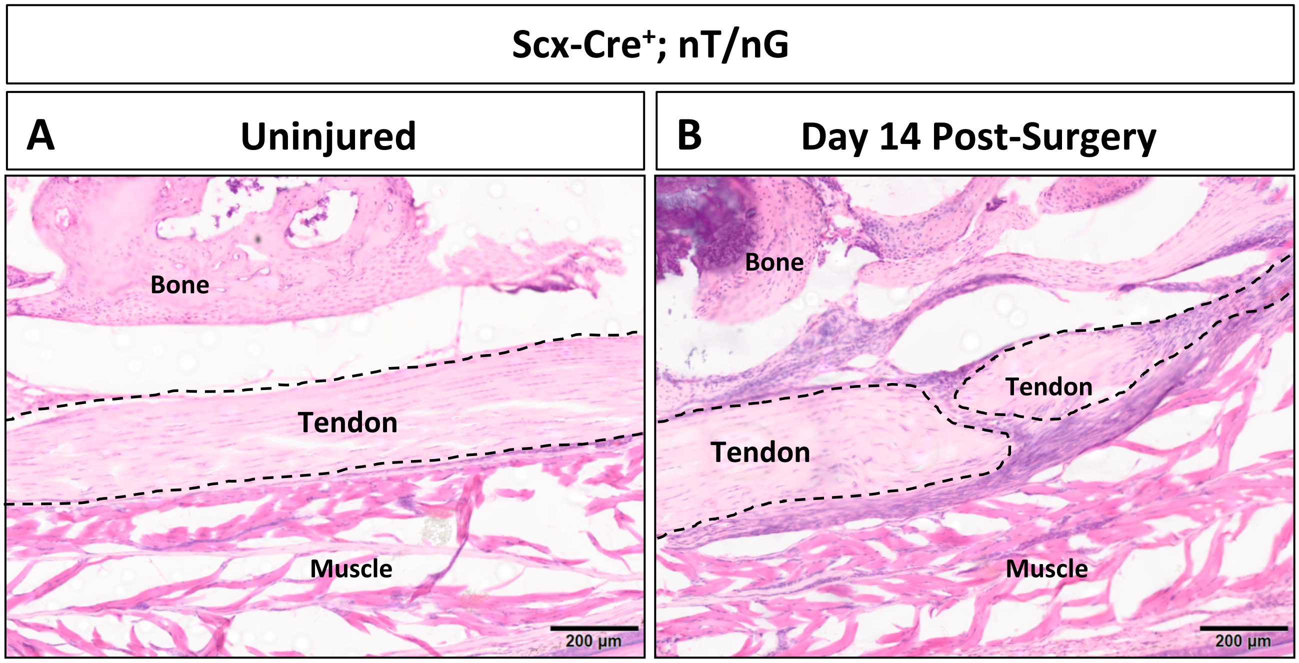

Supplemental Figure 2. Hematoxylin & Eosin (H & E) staining of (A) un-injured tendon and (B) day 14 post-surgery from Scx-Cre^{+}; nT/nG mice. Following fluorescent imaging (Figure 6), coverslips were gently removed and sections were stained with H&E to assess tissue and cell morphology. Tendon is outlined in black. Scale bars represent 200 microns.

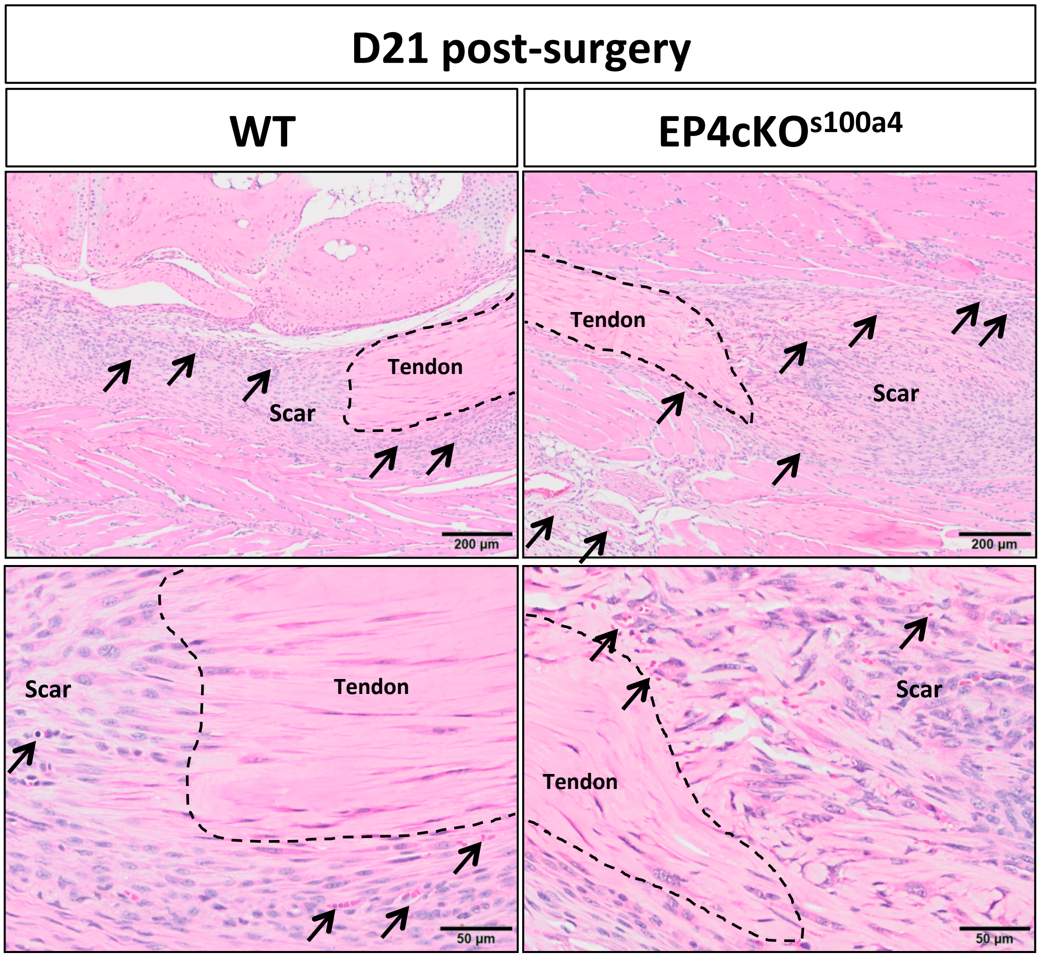

Supplemental Figure 3. Hematoxylin & Eosin (H & E) staining of (A & A') WT and (B & B') EP4cKO^{S100a4} tendon repairs at day 21 post-surgery. Following fluorescent imaging (Figure 7), coverslips were gently removed and sections were stained with H&E to assess tissue and cell morphology. Tendon is outlined in black. Scale bars represent 200 microns (A & B), or 50 microns (A' & B'). Blood vessels are identified by black arrows.

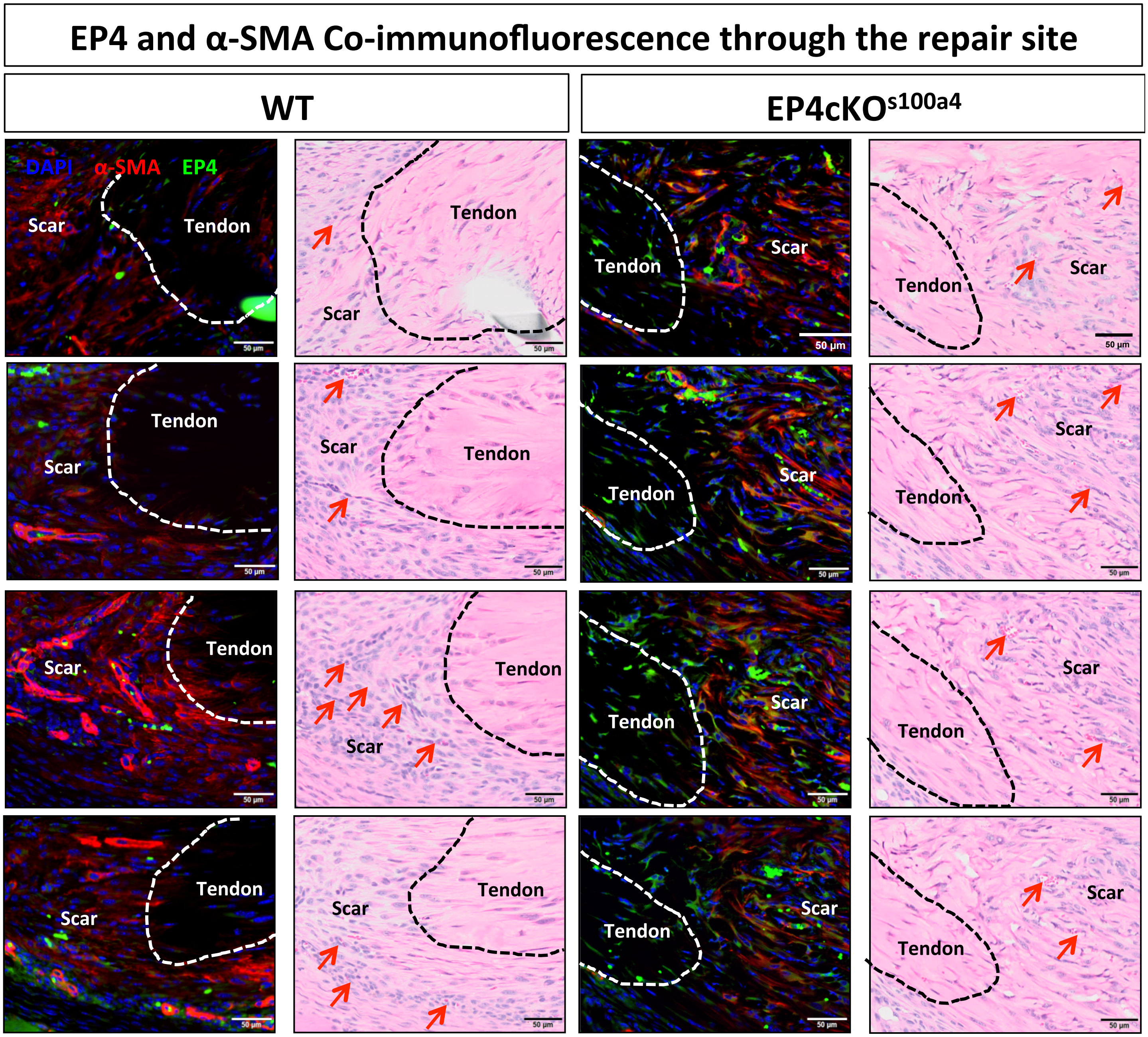

Supplemental Figure 4. To assess changes in α-SMA and EP4 protein expression through the depth of the repair site between WT and EP4cKO^{S100a4} repairs, four sections that were approximately 40 microns apart (spanning 120 microns of healing tissue) underwent co-immunoflourescent staining for α-SMA (red) and EP4 (green). Nuclei are stained blue with DAPI. Scale bars represent 50 microns. Red arrows indicate blood vessels in the H & E images.

Gene	Forward Primer (5'-3')	Reverse Primer (5'-3')
Col3a1	ACG TAG ATG AAT TGG GAT GCA G	GGG TTG GGG CAG TCT AGT G
Col1a1	GCT CCT CTT AGG CAC T	CCA CGT CTC ACC ATT GGG G
Scx	TGG CCT CCA GCT ACA TTT CT	TGT CAC GGT CTT TGC TCA AC
α-SMA	GAG GCA CCA CTG AAC CCT AA	CAT CTC CAG AGT CCA GCA CA
EP4	TTC CGC TCG TGG TGC GAG TGT TC	GAG GTG GTG TCT GCT TGG GTC AG
β-actin	AGA TGT GGA TCA GCA AGC AG	GCG CAA GTT AGG TTT TGT CA

Supplemental Table 1: Primer Sequences
